# Graduation of Medical Students

**Published:** 1854-03

**Authors:** 


GRADUATION OF MEDICAL STUDENTS.—The course of Lectures for the present Session of 1853–54, of the Medical Department of Iowa University, closed on Wednesday, Feb. 8th, after a most pleasant and prosperous lecture term of four months. On Tuesday evening, Feb. 9, the graduating exercises took place, and were largely attended. The degree of M. D., was conferred in an impressive manner, by Prof. Sanborn, upon the following graduates :

| | | |
|---|---|---|
| FRANCIS BRADY, | Knoxville, | Iowa, |
| JAMES D. GRAY, | " | " |
| W. S. HULL, | Dudley, | " |
| W. S. MARSH, | West Point, | " |
| J. N. NORRIS, | Birmingham, | " |
| A. A. RAMSAY, | Albia, | " |
| W. A. ROUSSEAU, | Washington, | " |
| HENRY RODGERS, | Newton, | " |
| S. H. SAWYERS, | Iconium, | " |
| W. B. SMITH, | Lancaster, | " |
| JOSEPH TALBOT, | Birmingham, | " |
| L. M. TIMMONS, | Knoxville, | " |
| N. VAN PATTEN, | De Witt, | " |

Honorary degrees were conferred upon—

| | | |
|---|---|---|
| Dr. A. HARD, | Marseilles, | Illinois, |
| Dr. RUBEY, | Clark Co., | Mo. |

After the ceremony of conferring the Degrees, a very instructive and appropriate address to the Graduting Class was delivered by Prof. Arnold, who was followed by Prof. M'Gugin, in a brief but excellent review of the past history of the Institution, with an encouraging statement of its present condition and prospects. We are happy to be able to record the pleasant termination of a very harmonious and fully attended course of Lectures, and have some pride in sending forth into the State a band of Physicians, as well prepared for their responsible labors as are those above enumerated. We have every reason to anticipate, at the next Session, a much larger Class than that of the last, and we have no higher wish than that the mutual gratification of Faculty and students shall equal that acknowledged during the session lately closed. In connection with this, we take pleasure in announcing that the Library, connected with the College, the nucleus of which was lately formed, has recently received large additions by generous private donations, but more particularly by the bestowment of all the publications of the Smithsonian Institute, at Washington, an accession exceedingly valuable in itself, and also from the fact, that it is the only copy of the same works in this vicinity, and will therefore be peculiarly useful, as a standard means of reference. The works have been obtained by the exertions and influence of Prof. M'Gugin, and they constitute one of the most valuable accessions to its resources the Institution has received.

